# Characteristics of psychiatric patients with nightmares after suvorexant administration: A retrospective study

**DOI:** 10.1002/npr2.12506

**Published:** 2024-11-29

**Authors:** Kazuya Yasuda, Yoji Hirano, Ryuichiro Takeda, Ryuji Ikeda, Yasushi Ishida

**Affiliations:** ^1^ Department of Pharmacy University of Miyazaki Hospital Miyazaki Japan; ^2^ Division of Clinical Neuroscience, Department of Psychiatry, Faculty of Medicine University of Miyazaki Miyazaki Japan; ^3^ Health Care and Safety Center University of Miyazaki Miyazaki Japan; ^4^ Nozaki Hospital Miyazaki Japan

**Keywords:** age, nightmare, psychiatric disorders, risk factors, suvorexant

## Abstract

**Aim:**

Suvorexant is an orexin receptor antagonist (ORA) for the treatment of insomnia. The antagonistic action of suvorexant on orexin receptors is associated with an increase in rapid eye movement (REM) sleep, which can potentially lead to nightmares depending on the patient's condition. However, the precise risk factors for nightmares among patients taking ORAs, such as suvorexant, have yet to be identified. In this retrospective study, we aimed to identify the risk factors for the development of nightmares in patients treated with suvorexant.

**Methods:**

The risk factors were determined by comparing parameters between the nightmare group and the nonnightmare group. This study included 440 patients who received suvorexant at the University of Miyazaki Hospital from April 2014 to January 2021.

**Results:**

We found that 9.1% (*n* = 40) of the patients experienced suvorexant‐induced nightmares. There was a significant difference in the median age, which was lower in the nightmare group than in the nonnightmare group (*p* < 0.01). Furthermore, both multiple logistic regression analysis and Cox proportional hazards regression analysis revealed increased odds ratios for nightmares for individuals aged 20–39 years.

**Conclusions:**

This study revealed that elderly patients taking suvorexant had fewer nightmares than nonelderly patients did.

## Abstract 2

This study investigates the risk factors for nightmares among psychiatric patients treated with suvorexant, an orexin receptor antagonist. A retrospective analysis of 440 patients revealed that 9.1% experienced nightmares, with younger patients (aged 20–39 years) being at a significantly higher risk. These findings suggest age‐dependent susceptibility to suvorexant‐induced nightmares, highlighting the importance of caution when prescribing this medication to younger individuals.

## Graphical abstract

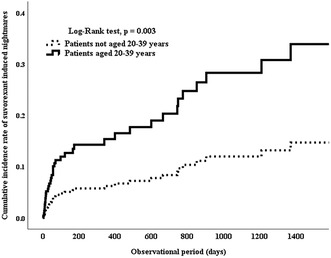

## 1 INTRODUCTION

Approximately one‐third of the general population presents with symptoms of insomnia.^1^ Persistent sleep disturbances, or insomnia, can exert considerable effects on an individual's mental condition, leading to an increased risk of developing several psychiatric disorders.^2^ In particular, insomnia can significantly promote the development and exacerbation of psychiatric disorders, such as anxiety disorders, depression and psychosis.^3^ Therefore, early intervention and appropriate treatment for insomnia are crucial.

With respect to the pharmacological treatment for insomnia, benzodiazepine receptor agonists (BRAs; benzodiazepines and nonbenzodiazepines) exert hypnotic effects by acting on γ‐aminobutyric acid type A (GABA_A_) receptors to suppress central nervous system activity and have been used to treat sleep disorders for many years. However, GABA_A_ receptors are widely localized in the brain, and BRAs can cause rebound insomnia, withdrawal symptoms, anterograde amnesia, muscle relaxation, falls and fractures among elderly individuals and individuals with respiratory depression.^4^, ^5^, ^6^ Furthermore, the development of tolerance and the potential for the abuse of BRAs have been widely documented over the years.^7^, ^8^ Consequently, health authorities in numerous countries, including Japan, have implemented guidelines advocating exclusively for short‐term treatment when prescribing BRAs.^9^, ^10^

In recent years, hypnotics that do not involve benzodiazepine receptors, such as melatonin receptor agonists and orexin receptor antagonists (ORAs), have also begun to be used. The advent of two innovative categories of hypnotic agents, namely, melatonin receptor agonists and ORAs (e.g., suvorexant and lemborexant), has heralded a transformative shift in the therapeutic paradigm for insomnia. In particular, the use of ORAs has increased since the approval of suvorexant in Japan.^11^ Suvorexant, the first selective dual ORA, suppresses excessive wakefulness by inhibiting orexin receptors that control wakefulness, thus leading to more physiological sleep.^12^

In contrast to BRAs, suvorexant facilitates both nonrapid eye movement (NREM) and rapid eye movement (REM) sleep, thereby preserving sleep‐stage‐specific quantitative electroencephalogram spectral profiles and affording somnolence indistinguishable from that of natural sleep.^13^ Given the established association between dreaming and REM sleep, which is characterized by longer, vivid, narrative, and bizarre dream experiences in contrast to NREM sleep,^14^, ^15^, ^16^ an augmented REM sleep duration could increase the incidence of nightmares.

This effect may vary on the basis of a patient's psychological predisposition and could disrupt the continuity of treatment. Nevertheless, no previous studies have examined the risk factors for suvorexant‐induced nightmares. While suvorexant has indeed exhibited efficacy in managing insomnia, if its discontinuation becomes necessary due to nightmares, patients may encounter several drawbacks, ultimately impacting their quality of life. BRAs have long been widely used in the treatment of insomnia, and many patients are still taking them. There are two methods of treating patients taking BRAs with suvorexant: continuing the BRA alongside suvorexant or substituting suvorexant for the BRA. It would be very beneficial to clarify which method is better for using suvorexant safely. Additionally, suvorexant is often used in elderly people at risk of delirium to exploit its antidelirium effects. We have concerns regarding whether suvorexant is suitable for elderly people. Identifying the risk factors for suvorexant‐induced nightmares can help in choosing the appropriate treatment.

Therefore, we retrospectively collected data from electronic medical records and compared patients with and without suvorexant‐induced nightmares to identify the risk factors for suvorexant‐induced nightmares.

## 2 METHODS

### 2.1 Participants

We conducted a retrospective analysis of patients treated for psychiatric disorders at the Department of Psychiatry, University of Miyazaki Hospital, who were administered suvorexant as a therapeutic intervention targeting insomnia. From November 2014 to January 2021, 605 patients were administered suvorexant treatment in our hospital's psychiatry department. A total of 440 patients were included. The exclusion criteria were as follows: an observation period of less than 2 weeks (80 patients) and a lack of essential data at treatment initiation (85 patients), especially among patients who were prescribed suvorexant by a doctor other than the psychiatrist at our hospital. The study period was from the start of suvorexant administration to either the discontinuation date or the last traceable date of the suvorexant treatment (within March 2023).

On the basis of electronic medical records prepared by the attending physician, the patients who discontinued suvorexant due to persistent and unmanageable nightmares between the start and end of suvorexant administration were classified into the “nightmare group”, and the others were classified into the “nonnightmare group”. The primary endpoint of this study was the incidence of nightmares leading to the discontinuation of suvorexant.

To identify factors influencing suvorexant‐induced nightmares, we analyzed the following factors before suvorexant administration: sex, age, white blood cell (WBC; 10^3^/μL) count, hemoglobin (Hb; g/dL) level, platelet (PLT; 10^3^/μL) count, total protein (TP; g/dL) level, albumin (ALB; g/dL) level, creatinine (CRE; mg/dL) level, total bilirubin (TB; mg/dL) level, glucose (GLU; mg/dL) level, triglyceride (TG; mg/dL) level, sodium (Na; mmol/L) level, potassium (K; mmol/L) level, aspartate aminotransferase (AST; U/L) level, alanine aminotransferase (ALT; U/L) level, gamma‐glutamyl transpeptidase (γGT; U/L) level, estimated glomerular filtration rate (eGFR; mL/min/1.73 m^2^), and C‐reactive protein (CRP; mg/dL) level. Furthermore, we compared the rates of persistent nightmares related to suvorexant between patients with and without a psychiatric diagnosis on the basis of the International Classification of Diseases, Tenth Revision (ICD‐10) diagnostic criteria. We analyzed the concomitant use of the following medications that were statistically useful: olanzapine, quetiapine, trazodone, aripiprazole/brexpirazole, selective serotonin reuptake inhibitors (SSRIs)/serotonin norepinephrine reuptake inhibitors (SNRIs), mirtazapine, risperidone/paliperidone, valproic acid, and lithium.

To verify the effects on nightmares induced by GABA_A_ receptor modulators, an investigation into the coadministration of BRAs, along with their respective utilization preceding the administration of suvorexant, was conducted. The switch group was defined as the group in which all or some of the BRAs were switched to suvorexant. The addition group was defined as the group for which suvorexant treatment was initiated without discontinuing or reducing the dose of BRAs. Patients who took suvorexant in combination with BRAs and whose BRA dose was subsequently reduced were included in the addition group.

### 2.2 Statistical analysis

For continuous data, the Mann–Whitney *U* test was used to compare the median values between the two groups. We used the chi‐square test or Fisher's exact test for nominal variable data. To analyze the odds ratios (ORs) of risk factors for the development of nightmares, multiple logistic regression analysis was performed.

Cox proportional hazards regression analysis and the Log‐Rank test were used to estimate and compare the cumulative incidence of suvorexant induced nightmares between the two groups. In the nightmare group, the period from the initiation of suvorexant administration to the onset of nightmares was plotted. In the non‐nightmare group, the period from the start of suvorexant administration at the hospital to the end of administration was plotted as censoring. The Hosmer–Lemeshow test for the logistic regression analysis revealed no significant difference (*p* = 0.9), and the area under the ROC curve (AUC) was 0.617.

The number of missing values for each parameter was as follows: WBC count, Hb level, PLT count, AST level, ALT level (*n* = 66), CRE level (*n* = 68), Na level, K level (*n* = 69), TP level, T‐BIL level (*n* = 79), ALB level (*n* = 83), GLU level (*n* = 96), γGT level (*n* = 101), CRP level (*n* = 150), TG level (*n* = 216), and eGFR (*n* = 258). Missing data were not imputed in this study. In this study, we used Cox proportional hazards regression analysis to analyze the time to nightmare onset, which was defined as the time from the start of suvorexant treatment to the onset of nightmares, taking censoring into account. The statistical analysis was performed via SPSS version 23, and the significance level was set to less than 5%.

### 2.3 Ethics statement

This study was approved (Approval No. O‐0951) by the Ethics Review Committee of the University of Miyazaki Hospital per the ethical guidelines for medical research for humans, and the protocol complied with the Declaration of Helsinki. The requirement for informed consent was waived owing to the retrospective nature of the study. The details of the study were described on a web page that patients could access from the hospital's website. If patients did not want to complete the study, they could inform us of their intentions.

## 3 RESULTS

We included 440 patients in this study, and the median period of suvorexant administration was 142 days (range: 3–2637 days). Among the 440 patients who initiated suvorexant treatment, the outcomes during the observation period were as follows: transferred to another hospital, 42.1% (*n* = 185); discontinued suvorexant treatment, 48.9% (*n* = 215); continued suvorexant treatment, 6.1% (*n* = 27); completed suvorexant treatment, 2.1% (*n* = 9); and death, 0.9% (*n* = 4). The reasons for discontinuing suvorexant were as follows: improvement in insomnia, 12.5% (*n* = 55); insufficient hypnotic effects, 11.8% (*n* = 52); nightmares, 9.1% (*n* = 40); hang‐over effects or excessive sedation, 7.5% (*n* = 33); no record, 5.0% (*n* = 22); drug interactions, 1.8% (*n* = 8); and other reasons, 1.1% (n = 5).

When the detailed suvorexant interruption factors due to nightmares were investigated, we found a significant difference in the median age (*p* = 0.015) and median eGFR (*p* = 0.027) between patients with and without nightmares after suvorexant administration. Analysis of the categorized factors revealed significant differences in patients aged 20–39 years (*p* = 0.007) (Table 1). Based on the results of the univariate analysis, we selected the median age, age group 20–39 years, eGFR, switch from a BRA, and BRA use. We then examined correlations among these variables to identify and exclude potential confounding factors. The variance inflation factor (VIF) was calculated for each key variable prior to logistic regression analysis: age (VIF: 2.841), an age of 20–39 years (VIF: 2.213), eGFR (VIF:1.488), BRA use (VIF: 1.018), and switch from a BRA (VIF: 1.016). The calculation of Pearson's correlation coefficients revealed significant correlations between age, an age of 20–39 years and eGFR (*p* < 0.0001). Because suvorexant is not excreted primarily through the kidney and most of the eGFR data were missing, an age of 20–39 years was selected from these correlated factors.

**TABLE 1:** Comparisons of each risk factor in the exposed group.

	All patients	Nightmare (−)	Nightmare (+)	*p* value
Number of patients	440	400	40	
Sex, *n* (%)				
Male	171 (38.9)	158 (39.5)	13 (32.5)	0.386^a^
Age, years, Median (range)	53 (12–90)	54 (12–90)	44 (16–80)	0.015^*,^ ^b^
Age Group, *n* (%)				
0–19 y.o.	18 (4.1)	16 (4.0)	2 (5.0)	0.674^c^
20–39 y.o.	101 (23.0)	85 (21.3)	16 (40.0)	0.007^*,^ ^a^
40–59 y.o.	146 (33.2)	134 (33.5)	12 (30.0)	0.654^a^
60–79 y.o.	138 (31.4)	130 (32.5)	8 (20.0)	0.111^c^
80 and over.	37 (8.4)	35 (8.8)	2 (5.0)	0.560^c^
Laboratory data, Median (range)				
WBC, 10^3^/μL	6.00 (2.5–15.10)	6.00 (2.50–15.10)	5.95 (3.60–11.70)	0.782^b^
Hb, g/dL	13.1 (6.3–18.20)	13.1 (6.30–18.20)	13.2 (10.20–17.10)	0.482^b^
PLT, 10^3^/μL	227 (13.2–518)	227 (13–518)	248 (177–451)	0.191^b^
TP, g/dL	6.76 (3.95–8.4)	6.76 (3.95–8.40)	6.74 (5.74–8.00)	0.900^b^
ALB, g/dL	4.01 (1.82–5.13)	4.00 (1.82–5.13)	4.06 (2.77–4.93)	0.438^b^
CRE, mg/dL	0.68 (0.30–6.07)	0.68 (0.30–6.07)	0.58 (0.34–1.30)	0.134^b^
T‐BIL, mg/dL	0.60 (0.20–7.80)	0.60 (0.20–7.80)	0.75 (0.30–1.30)	0.151^b^
GLU, mg/dL	94 (52–687)	95 (52–687)	91 (66–117)	0.185^b^
TG, mg/dL	100 (30–808)	97 (30–808)	123 (49–339)	0.180^b^
Na, mmol/L	140 (102–158)	140 (125–158)	140 (102–142)	0.953^b^
K, mmol/L	4.0 (2.7–5.8)	4.0 (2.7–5.8)	3.9 (3.2–5.0)	0.305^*,^ ^b^
AST, mmol/L	20 (4–263)	20 (4–263)	21 (12–62)	0.709^b^
ALT, mmol/L	16 (3–411)	16 (3–411)	18 (5–93)	0.556^b^
γGT, U/L	21 (4–1288)	21 (5–1288)	22 (4–103)	0.625^b^
eGFR, mL/min/1.73m^2^	77.4 (7.8–201.2)	76.8 (7.8–201.2)	88.3 (50.6–173.0)	0.027^*,^ ^b^
CRP, mg/dL	0.06 (0.00–12.13)	0.06 (0.00–12.13)	0.07 (0.01–3.51)	0.713^b^
ICD‐10, *n* (%)				
F00–F09	113 (25.7)	100 (25.0)	13 (32.5)	0.301^a^
F10–F19	27 (6.1)	26 (6.5)	1 (2.5)	0.273^c^
F20–F29	207 (47.0)	185 (46.3)	22 (55.0)	0.290^a^
F30–F39	258 (58.6)	235 (58.8)	23 (57.5)	0.878^a^
F40–F48	195 (44.3)	179 (44.8)	16 (40.0)	0.564^a^
F50–F59	15 (3.4)	12 (3.0)	3 (7.5)	0.147^c^
F60–F69	11 (2.5)	9 (2.3)	2 (5.0)	0.263^c^
F70–F79	19 (4.3)	18 (4.5)	1 (2.5)	1.000^c^
F80–F89	15 (3.4)	15 (3.8)	0 (0.0)	0.381^c^
F90–F98	2 (0.5)	1 (0.3)	1 (2.5)	0.174^c^
Coadministration, *n* (%)				
Olanzapine	41 (9.3)	35 (8.8)	6 (15.0)	0.246^c^
Quetiapine	29 (6.6)	29 (7.2)	0 (0.0)	0.095^c^
Trazodone	12 (2.7)	11 (2.8)	1 (2.5)	1.000^c^
Aripiprazole or brexpiprazole	53 (12.0)	46 (11.5)	7 (17.5)	0.304^c^
SSRI or SNRI	88 (20.0)	82 (20.5)	6 (15.0)	0.535^c^
Mirtazapine	29 (6.6)	24 (6.0)	5 (12.5)	0.168^c^
Risperidone or paliperidone	18 (4.1)	17 (4.3)	1 (2.5)	1.000^c^
Valproic acid	31 (7.0)	30 (7.5)	1 (2.5)	0.341^c^
Lithium	10 (2.3)	10 (2.5)	0 (0.0)	0.610^c^
Benzodiazepine	109 (24.8)	96 (24.0)	13 (32.5)	0.235^a^
Nonbenzodiazepine	77 (17.5)	74 (18.5)	3 (7.5)	0.123^c^
BRA	166 (37.7)	152 (38.0)	14 (35.0)	0.709^a^
Switch from a benzodiazepine	65 (14.8)	57 (14.2)	8 (20.0)	0.349^c^
Switch from a nonbenzodiazepine	36 (8.2)	33 (8.3)	3 (7.5)	1.000^c^
Switch from a BRA	100 (22.7)	89 (22.3)	11 (27.5)	0.450^a^

*Note*: Data are presented as the number of patients (mean ± SD) or median (range).

F00–F09: organic, symptomatic mental disorders. F10–F19: mental and behavioral disorders due to psychoactive substance use. F20–F29: schizophrenia, schizotypal, and delusional disorders. F30–F39: mood [affective] disorders. F40–F48: neurotic, stress‐related, and somatoform disorders. F50–F59: behavioral syndromes associated with physiological disturbances and physical factors. F60–F69: Disorders of adult personality and behavior. F70–F79: mental retardation. F80–F89: disorders of psychological development. F90–F98: behavioral and emotional disorders with onset usually occurring in childhood and adolescence.

Abbreviations: ALB, albumin; ALT, alanine aminotransferase; AST, aspartate aminotransferase; BRAs, benzodiazepine receptor agonists (benzodiazepines and nonbenzodiazepines); CRE, creatinine; CRP, C‐reactive protein; eGFR, estimated glomerular filtration rate; GLU, glucose; Hb, hemoglobin; ICD‐10, International Classification of Diseases, Tenth Revision; K, potassium; Na, sodium; PLT, platelet; SD, standard deviation; SNRIs, serotonin norepinephrine reuptake inhibitors; SSRIs, selective serotonin reuptake inhibitors; TB, total bilirubin; TG, triglyceride; TP, total protein; WBC, white blood cell; γGT, γ‐glutamyl transpeptidase.

^a^ *χ* ^2^ test.

^b^ Mann–Whitney *U* test.

^c^ Fisher's exact test.

*p < 0.05.

On the basis of these results, we performed a logistic regression analysis. In the logistic regression analysis, an age of 20–39 years (OR = 2.471, 95% CI = 1.256–4.859, *p* = 0.009) was identified as a risk factor. Cox proportional hazards regression analysis was conducted, with age 20–39 years, concomitant BRA use, and switch from a BRA included as independent variables. The analysis revealed that age 20–39 years was significantly associated with the cumulative incidence of nightmare onset related to suvorexant use (hazard ratio: 2.64; 95% CI: 1.392–5.005; *p* = 0.003). There was no significant difference between switch from a BRA and using BRA (Table 2).

**TABLE 2:** Multiple analyses of suvorexant induced nightmare onset.

Logistic regression analysis	Odds ratio	95% CI	*p* value
20–39 y.o.	2.512	1.273–4.960	0.008*
Coadministration of BRA	0.924	0.464–1.837	0.821
Switch from a BRA	1.407	0.670–2.955	0.368

Cox proportional hazards regression analysis	Hazard ratio	95% CI	*p* value
20–39 y.o.	2.64	1.392–5.005	0.003*
Coadministration of BRA	0.69	0.358–1.33	0.268
Switch from a BRA	1.362	0.677–2.739	0.386

Abbreviations: CI, confidence interval.

**FIGURE 1:**
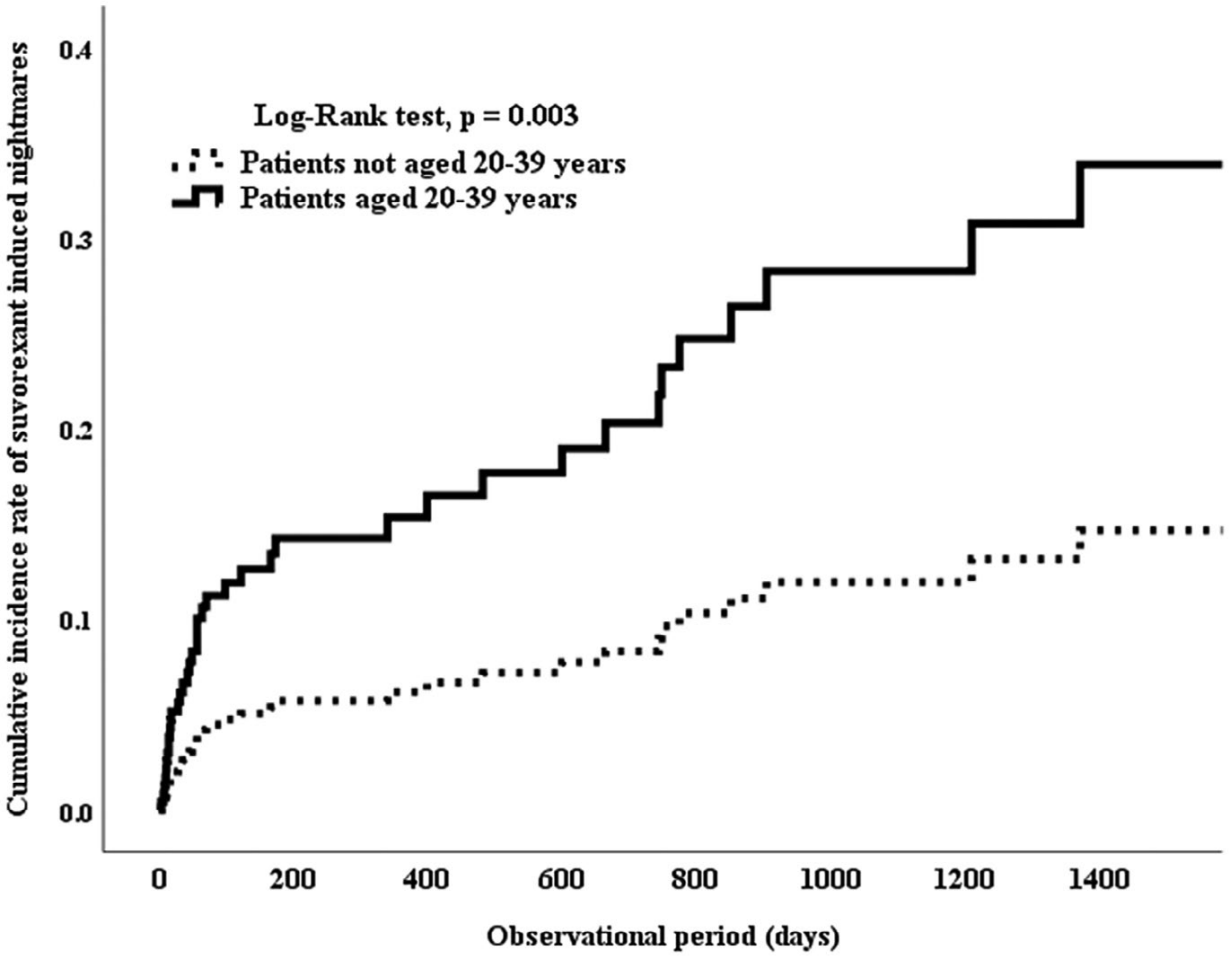
Effects of age 20–39 years on the cumulative incidence of suvorexant induced nightmares. Solid line: Patients aged 20–39 years. Dotted line: Patients not aged 20–39 years. A significant difference was observed between the two groups according to the log‐rank test (*p* = 0.003).

In terms of the cumulative incidence of suvorexant induced nightmares, we found that patients aged 20–39 years experienced an earlier onset of nightmares compared to those outside this age group (*p* = 0.003) (Figure 1).

## 4 DISCUSSION

In this study, we elucidated the risk factors for suvorexant‐induced nightmares. Briefly, we found the following: (1) 9.1% (*n* = 40) of the patients experienced nightmares that made it impossible to continue taking suvorexant; and (2) elderly individuals had fewer nightmares when taking suvorexant than nonelderly individuals did.

Nightmares can cause daytime deterioration.^17^ Additionally, nightmares are associated with suicidal ideation^18^ and increase the risk of suicide.^19^ A study of middle‐aged individuals reported that traits such as low socioeconomic status, insomnia, poor sleep quality, and neurotic personality traits were associated with nightmares.^20^ Thus, early diagnosis and interventions are critical.

While suvorexant is very effective for the treatment of insomnia, very vivid and terrifying nightmares are frequently reported by patients taking suvorexant.^21^ A meta‐analysis revealed that compared with a placebo, suvorexant increased the frequency of nightmares, with a relative risk of 2.08%.^22^ Moreover, the incidence of nightmares in patients taking suvorexant in the postmarketing safety population was 0.8%,^23^ and 6.67% of the 30 adolescents who took suvorexant had nightmares that made continuing treatment difficult.^24^ In our study, 9.1% of the patients experienced suvorexant‐induced nightmares, leading to discontinuation of the medication. Despite differences in the study population, such as different psychiatric settings and age distributions, the incidence rates were approximately the same.

Importantly, in this study, we identified younger age as a risk factor for nightmares in patients treated with suvorexant. Specifically, this study revealed significant differences in the incidence of suvorexant‐induced nightmares in the 20–39 years age group. Therefore, adequate information regarding the potential risk of nightmares is crucial when prescribing suvorexant to patients in this age group. The rhythm and quality of sleep change with age. For example, in elderly individuals, the circadian rhythm advances,^25^ resulting in heightened arousal during sleep, accompanied by diminished occurrence of wave‐breaking sleep and sleep spindle activity.^25^, ^26^, ^27^, ^28^ Previous studies have reported decreased REM sleep in elderly individuals.^29^, ^30^ Consequently, given that dreams, encompassing nightmares, transpire during REM sleep, it is posited that older age has a relatively attenuated effect on suvorexant‐induced nightmares. Conversely, the incidence of nightmares is highest during late adolescence and early adulthood.^31^ Moreover, detailed studies examining the prevalence of nightmares at all ages have shown that the prevalence of nightmares is lower in older adults than in younger adults.^32^, ^33^, ^34^

Drugs other than suvorexant affect the incidence of dreams and nightmares. Specifically, it has been noted that trazodone has a suppressive effect on nightmares in patients with advanced cancer.^35^ Additionally, pharmaceutical agents that impact neurotransmitter activity within the central nervous system, such as antidepressants and antihypertensives, have been linked to the manifestation of nightmares.^36^, ^37^ Furthermore, a previous report suggested that concomitant quetiapine use might contribute to the continuation of suvorexant.^38^ Thus, in this study, the impact of the concurrent use of these medications on suvorexant‐induced nightmares was examined, but no particularly significant findings were obtained. This is likely because the study analyzed a limited number of patients at a single institution, resulting in few instances of concomitant drug use. A meta‐analysis revealed that women tend to report nightmares more often than men do.^39^ However, our study did not reveal any sex differences in the risk of suvorexant‐induced nightmares. In other words, suvorexant‐induced nightmares were indicated to possess distinctive characteristics compared with typical nightmares, with no discernible sex‐based differences.

Since BRA suppresses REM sleep,^40^, ^41^ it was hypothesized that switching from a BRA to suvorexant or the concurrent use of a BRA at the time of suvorexant initiation would affect the incidence of nightmares. However, no significant differences were observed in this study. The results provide reassuring data for promptly switching from a BRA to an ORA. The impact of switching from or adding a BRA on the incidence of nightmares is considered minimal.

This study has several limitations. First, this was a single‐center study with a limited number of cases. The sample size may not be sufficient to analyze detailed differences. Second, this study is cross‐sectional and does not follow the same subjects longitudinally. Therefore, for more detailed validation, longitudinal studies are desirable in the future. This study was retrospective and did not have fixed evaluators. In other words, the decision to discontinue medication due to nightmares was made by different psychiatrists. Thus, for more rigorous validation, it will be necessary to either have a single evaluator or establish strict, unified criteria for medication discontinuation. Third, suvorexant use began in November 2014, and the study observed suvorexant use until 2023. Therefore, the increased experience and knowledge of suvorexant use by psychiatrists during the study period may have influenced the incidence of suvorexant discontinuation due to nightmares. Fourth, because this study investigated severe nightmares that led to the discontinuation of suvorexant, the effects of age cannot be ruled out, as psychiatrists may want to reduce the use of sleeping pills as much as possible in younger patients (people who are working, students, and have opportunities to study). Fifth, in this study, the area under the ROC curve (AUC) in the logistic regression analysis was 0.617, which is not high, thus slightly reducing the validity of the logistic regression analysis. A multicenter prospective study with more cases should be conducted in the future.

Importantly, recent insights from various reports have consistently demonstrated that the use of suvorexant, a sleep aid, results in a favorable balance between safety and efficacy for elderly individuals, as evidenced by the findings of this study. Specifically, suvorexant is reported to be well tolerated in older adults with insomnia^42^ and may reduce the risk of falls.^43^ It also inhibits delirium, which occurs at high rates in elderly individuals.^44^

In conclusion, this study revealed that the prevalence of nightmares caused by suvorexant was significantly greater in those aged 20–39 years. Considering the negligible influence of nightmares on elderly individuals, our study emphasizes the user‐friendly nature of suvorexant as a convenient pharmacological intervention for addressing insomnia, especially in the elderly population.

## CONFLICT OF INTEREST

There are no conflicts of interest to disclose.

## APPROVAL OF THE RESEARCH PROTOCOL BY AN INSTITUTIONAL REVIEWER BOARD

This study was approved by the Institutional Review Board of the University of Miyazaki. The approval number was O‐0951, which was assigned in February 2022.

## INFORMED CONSENT

The requirement for informed consent was waived because of the retrospective nature of the study. The details of the study were described on a web page that patients could access from the hospital's website. If patients did not want to complete the study, they can inform us of their intentions.

## REGISTRY AND THE REGISTRATION NO. OF THE STUDY/TRIAL

N/a.

## Data Availability

We cannot provide the raw data to the public. The disclosure of personal data was not mentioned in the research protocol approved by the institutional review board.
